# Knowledge, Attitude, Practice and Barriers on Vaccination against Human Papillomavirus Infection: A Cross-Sectional Study among Primary Care Physicians in Hong Kong

**DOI:** 10.1371/journal.pone.0071827

**Published:** 2013-08-21

**Authors:** Martin C. S. Wong, Albert Lee, Karry L. K. Ngai, Josette C. Y. Chor, Paul K. S. Chan

**Affiliations:** 1 School of Public Health and Primary Care, Faculty of Medicine, The Chinese University of Hong Kong, Hong Kong Special Administrative Region, People’s Republic of China; 2 Department of Microbiology, Faculty of Medicine, The Chinese University of Hong Kong, Hong Kong Special Administrative Region, People’s Republic of China; Chinese Academy of Medical Sciences, China

## Abstract

This study explored the knowledge, attitude, practice and barriers to prescribe human papillomavirus (HPV) vaccines among private primary care physicians in Hong Kong. A self-administered questionnaire survey was conducted by sending letters to doctors who had joined a vaccination program for school girls. From 720 surveys sent, 444 (61.7%) completed questionnaires were returned and analyzed. For knowledge, few responded to questions accurately on the prevalence of cervical HPV (27.9%) and genital wart infection (13.1%) among sexually active young women in Hong Kong, and only 44.4% correctly answered the percentage of cervical cancers caused by HPV. For attitude, most agreed that HPV vaccination should be fully paid by the Government (68.3%) as an important public health strategy. Vaccination against HPV was perceived as more important than those for genital herpes (52.2%) and Chlamydia (50.1%) for adolescent health, and the majority selected adolescents aged 12–14 years as the ideal group for vaccination. Gardasil^®^ (30.9%) and Cervarix^®^ (28.0%) were almost equally preferred. For practice, the factors influencing the choice of vaccine included strength of vaccine protection (61.1%), long-lasting immunity (56.8%) and good antibody response (55.6%). The most significant barriers to prescribe HPV vaccines consisted of parental refusal due to safety concerns (48.2%), and their practice of advising vaccination was mostly affected by local Governmental recommendations (78.7%). A substantial proportion of physicians had recommended HPV vaccines for their female clients/patients aged 18–26 years for protection of cervical cancer (83.8%) or both cervical cancer and genital warts (85.5%). The knowledge on HPV infection was low among physicians in Hong Kong. Prescription of HPV vaccine was hindered by the perceived parental concerns and was mostly relied on Governmental recommendations. Educational initiatives should be targeted towards both physicians and parents, and the Government should consider full subsidy to enhance vaccine uptake rate.

## Introduction

Human papillomavirus (HPV) infection is the most common sexually transmitted disease worldwide. It has been estimated that at least 50% of sexually active people had acquired genital HPV infection during their lifetime [1], and in a recent report the prevalence of genital HPV infection was 14% in females aged 26–30 years [2]. Virtually all cases of cervical cancer are attributed to HPV, with HPV 16 and 18 accounting for 58% to 75% of all cervical cancers in Asia Pacific countries which was similar to Western countries [3]–[8]. Two vaccines (Gardasil®, Merck & Co. and Cervarix®, GlaxoSmithKline) have demonstrated high efficacy against cervical intraepithelial neoplasia and invasive cancers associated with vaccine-types (HPV16 and 18) [9]–[13], and their administration have been anticipated to be a crucial measure to reduce the incidence of cervical cancer worldwide [14], [15].

However, in order for the vaccination programmes to be successful, a high uptake rate is needed. Recommendation of HPV immunization by primary care physicians (PCPs) has been recognized as one the most influential factors in the individual’s willingness to receive the vaccine. Updated knowledge and positive attitude towards the vaccine among PCPs, as well as the removal of any barriers to prescription are the main determinant factors [16], [17]. PCPs represent a taskforce in the healthcare system which offered first-contact, coordinative preventive care to the general public and are in a privileged position to prescribe HPV immunizations. Many studies showed that physicians’ experience and attitudes towards HPV vaccine were major motivators affecting women and adolescent girls to receive the immunization [18]–[20].

Nevertheless, most of the existing studies were conducted among physicians in Western countries [21]–[29]. Half of the cervical cancer cases are in Asia [30], but there is a scarcity of original research conducted in Asia [31]. The differences in cultural environment and clinical practice among physicians practicing in different cultural and clinical environment remain to be explored.

The objectives of this study were to evaluate the knowledge on HPV infection, and the attitude towards and perceived barriers of HPV vaccination among PCPs in Hong Kong. Their practice of prescribing HPV vaccine according to recommendations and patient characteristics was also explored.

## Methods

### Sampling Frame and Recruitment Strategies

As HPV is not under universal coverage in Hong Kong, a Family-School-Doctor Model was developed by university academics in 2011 to improve the initiation of HPV vaccine among adolescent girls by tackling the socio-cultural barriers and also the barriers related to cost and convenience [32]. In Hong Kong, HPV vaccination is not provided in the public sector and patients have to pay out-of-pocket to receive the immunization in the private sector. Online education platform was built to provide free education videos for students and parents (http://www.youitv.com/partner/hps/cervicalcancer/). The contents of the online platform include the prevalence of cervical cancer, causes, symptoms, preventive measure and information of the vaccine. Schools were invited to join a special seminar conducted by the Chinese University of Hong Kong for knowledge enrichment. Schools were given an option to join the voluntary HPV vaccination programme, and participated students had the first dose (Cervarix®) free of charge on site at their schools. The acceptance of the 1^st^ free-of-charge dose of Cervarix at school was over 80%, and there has been no local recommendations regarding interchangeable use of two HPV vaccines. Physicians were not recommended to prescribe Gardasil after an initial dose of Cervarix. The second and third doses were then administered in the clinics of PCPs at market price determined by individual physicians. The PCPs in Hong Kong consist of general practitioners, family physicians, and specialists in the private sector who practice in the community setting including medical internists, gynecologists, pediatricians and oncologists. All 720 PCPs who participated in the school HPV vaccination programme received an invitation letter to complete an anonymous questionnaire designed for the current study.

Since part of this survey assessed the knowledge of the doctors on the research topic, the surveys were kept anonymous and did not require participants’ signatures. The completion of the survey by the study participants implied their consent. In terms of the measures that were taken to document the process, the reseachers invited the participants to return their completed surveys in an enclosed envelope after which they could sign their name in an attendance sheet to obtain Continuous Medical Education (CME) points. The return of their surveys was recorded to evaluate the response rate. The Survey and Behavioral Research Ethics Committee of The Chinese University of Hong Kong has approved this study and the consent procedure. The study did not involve minors/children participants, thus the concerns related to minors/children participants were not applicable in this study.

### Survey Instrument

The survey used in this study was designed by an academic physician, and validated by an expert panel consisting of epidemiologists, public health specialists, family physicians, microbiologists and academic professionals. The survey was pilot-tested by 20 physicians for face-validity and comprehensibility before study commencement. In addition, the information provided in the online platform was not related to the questions in the survey to ensure accurate assessment of the PCPs’ knowledge. We used a five-point Likert scale for survey items which assessed attitude towards the HPV vaccine. These questions are close-ended with possible responses including “extremely important”, “very important”, “important”, “somewhat important”, and “not at all”. The same applied to questions on their perceived barriers and likelihood to follow recommendations on administration of HPV vaccines. The questionnaire consists of 10 pages with 33 questions, and English was used in the survey as all PCPs in Hong Kong know English. For assessment of PCPs’ knowledge on HPV vaccine, the correctness of an answer was established from a thorough literature search and validation by the expert panel who designed the survey. For comparison of importance among different vaccines, one question item was phrased as “Compared to the following other vaccines that are in use or development, how would you rank the HPV vaccine in terms of importance for adolescent health?” According to our pilot study, the PCPs will need an average of 10 minutes to complete the questionnaire.

### Statistical Analysis

The Statistical Package for Social Sciences (SPSS, Chicago, IL) version 16.0 was used for all data entry and analyses. All descriptive statistics were presented as proportions. Chi square tests of heterogeneity were used to compare proportions. As an exploratory analysis, the proportions of physicians choosing Cervarix^®^ vs. Gardasil^®^ in their routine practice were compared according to the reasons of vaccine choice. P values <0.05 were regarded as statistically significant.

## Results

### Participant Characteristics

Of the 720 questionnaires distributed, 444 completed ones were collected (Table 1). The average age was 48.5 years (SD 12.1). 77.3% were male, and half (52.8%) had post-registration experience of more than 20 years (Table 1). The proportions of general practitioners or specialists in family medicine, pediatricians, and gynecologists were 76.4%, 12.2% and 4.3%, respectively. 44.8% of them reported seeing 26–99 patients aged between 10 to 17 years per week, and 42.1% saw 26–99 parents with teenage children per week. 82.2% indicated that they did not see any patients with cervical cancer in a typical month. In a typical week, 63.0% did not perform any Pap smears, and 34.1% reported performing 1–25 Pap smears. The majority of these physicians prescribed 1–25 HPV vaccines in the past 3 months for girls aged 10–17 years (77.8%), girls aged 18–26 years (84.3%), and women aged 27–45 years (74.0%). HPV vaccinations were rarely prescribed for older women, boys and men. Nearly 15% of PCPs never advised their adolescent patients on sex related matters and about two thirds advised in less than 25% of their consultation with adolescents (Table 1).

**Table 1:** Participant Characteristics (N = 444).

	N				%			
**Age** (mean +/− S.D.)	48.5				12.1			
**Gender**								
Female	98				22.1			
Male	343				77.3			
**Years after post registration**								
Over 30 years	117				26.4			
21 to 30 years	117				26.4			
11–20 years	147				33.1			
10 years or below	58				13.1			
**Clinical specialty**								
Pediatrician	54				12.2			
Gynecologist	19				4.3			
Oncologist	1				0.2			
Internal Medicine specialist	12				2.7			
General Practice/Family Medicine	339				76.4			
Others	14				3.2			
**Number of patients seen in a typical week**								
a). Aged 10–17 years								
None	14				3.2			
Jan-25	150				33.8			
26–99	199				44.8			
≥100	79				17.8			
b). Parents with teenage children								
None	16				3.6			
Jan-25	135				30.4			
26–99	187				42.1			
≥100	100				22.5			
**Number of patients with cervical cancer seen in a typical month**								
None	365				82.2			
Jan-25	66				14.9			
26–99	7				1.6			
≥100	1				0.2			
**Number of Pap smear performed in a typical week**								
None	277				63			
Jan-25	150				34.1			
26–99	11				2.5			
≥100	2				0.5			
**Number of HPV vaccines prescribed in the past 3 months**	**None**		**1–25**		**26–99**		**≥100**	
	**n**	**%**	**n**	**%**	**n**	**%**	**n**	**%**
*Girls 10–17 years*	86	21.0	318	77.8	4	1.0	1	0.2
*Girls 18–26 years*	56	13.3	354	84.3	7	1.7	3	0.7
*Women 27–45 years*	98	23.4	310	74.0	10	2.4	1	0.2
*Women >45 years*	327	87.9	43	11.6	1	0.3	1	0.3
*Boys 10–15 years*	356	95.4	15	4.0	1	0.3	1	0.3
*Boys/men >15 years*	357	94.9	17	4.5	0	0.0	2	0.5
**Frequency of discussion of sexual activity with adolescent patients during a usual visit**	**n**				**%**			
Never	61				13.9			
≤25% of visits	291				66.4			
25–50% of visits	45				10.3			
50–90% of visits	21				4.8			
>90% of visits	10				2.3			
Not applicable	10				2.3			

HPV: Human Papillomavirus.

### Knowledge of HPV Vaccination

Only 27.9% and 13.1% of physicians correctly responded to questions on the prevalence of cervical HPV infection and genital wart infection, respectively, among sexually active young women in Hong Kong (Table 2). Only 44.4% correctly answered the percentage of cervical cancers caused by HPV. However, high proportions of physicians could accurately identify the disease entities associated with HPV 6 and 11 (84.7%) and HPV 16 and 18 (88.1%).

**Table 2:** Knowledge on human papillomavirus (HPV) infection (N = 444).

	n	%	N (correct answers given)	% (correct answers given)
**Prevalence of HPV infection in sexually active young female in HK**				
5–10%	106	23.9	124	27.9
11–30%	124	27.9		
31–50%	71	16.0		
51–75%	54	12.2		
76–100%	18	4.1		
**Prevalence of genital warts infection in sexually active young female in HK**				
≤1%	58	13.1	58	13.1
2–5%	129	29.1		
6–10%	95	21.4		
11–20%	57	12.8		
>20%	31	7.0		
**Percentage of cervical cancer caused by infection with HPV**				
0–25%	37	8.3	197	44.4
26–50%	32	7.2		
51–75%	108	24.3		
76–100%	197	44.4		
**HPV subtypes 6 & 11 commonly associated with what disease**				
Genital warts	376	84.7	376	84.7
Plantar warts	4	0.9		
Cervical carcinoma	30	6.8		
Unsure	20	4.5		
**HPV subtypes 16 & 18 commonly associated with what disease**				
Genital warts	15	3.4	391	88.1
Plantar warts	4	0.9		
Cervical carcinoma	391	88.1		
Unsure	18	4.1		

### Attitudes towards Payment, Importance and Ideal Age Range of HPV Vaccination

**Figure 1:**
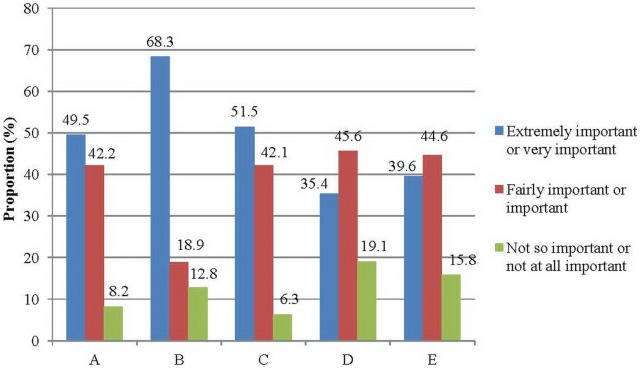
Attitude towards the importance of public health strategies to promote human papillomavirus vaccination. (A: Partial (∼30%) subsidy by the Government; B: Fully paid by the Government; C: Price offered at 50% discount; D: School immunization program with market price; E: Immunization program jointly organized by school and community doctors with market price).

The participants were asked about the importance of different public health strategies for promoting HPV vaccination (Fig. 1). They were of the view that HPV vaccination should be fully paid by the Government as an “extremely important” or “very important” public health strategy (68.3%). This was followed by “prices offered at a 50% discount” (51.5%) and “partial subsidy by the Government at a 30% discount” (49.5%). Immunization program jointly organized by school and community doctors with market price (39.6%), and school immunization programmes at market price (33.4%) were not regarded as important strategies. When the participants were asked whether HPV vaccination was more important than other vaccines for adolescent health, most agreed that HPV immunization was more important than those for genital herpes (52.2%) and chlamydia (50.1%). Many regarded HPV vaccination as equally important when compared with hepatitis B (61.7%), tuberculosis (57.4%), influenza (45.4%) and chickenpox (47.8%).

The attitudes of participating PCPs towards the ideal age range for HPV vaccine administration were explored. Most physicians indicated that 12–14 years (45.6%), 10–11 years (31.6%), and 15–17 years (20.3%) were the ideal age groups. Very few regarded ages 18 years or older as ideal for HPV vaccination.

### Preference and Reasons to Choose HPV Vaccines for Adolescent Girls Aged 10–17 Years

**Figure 2:**
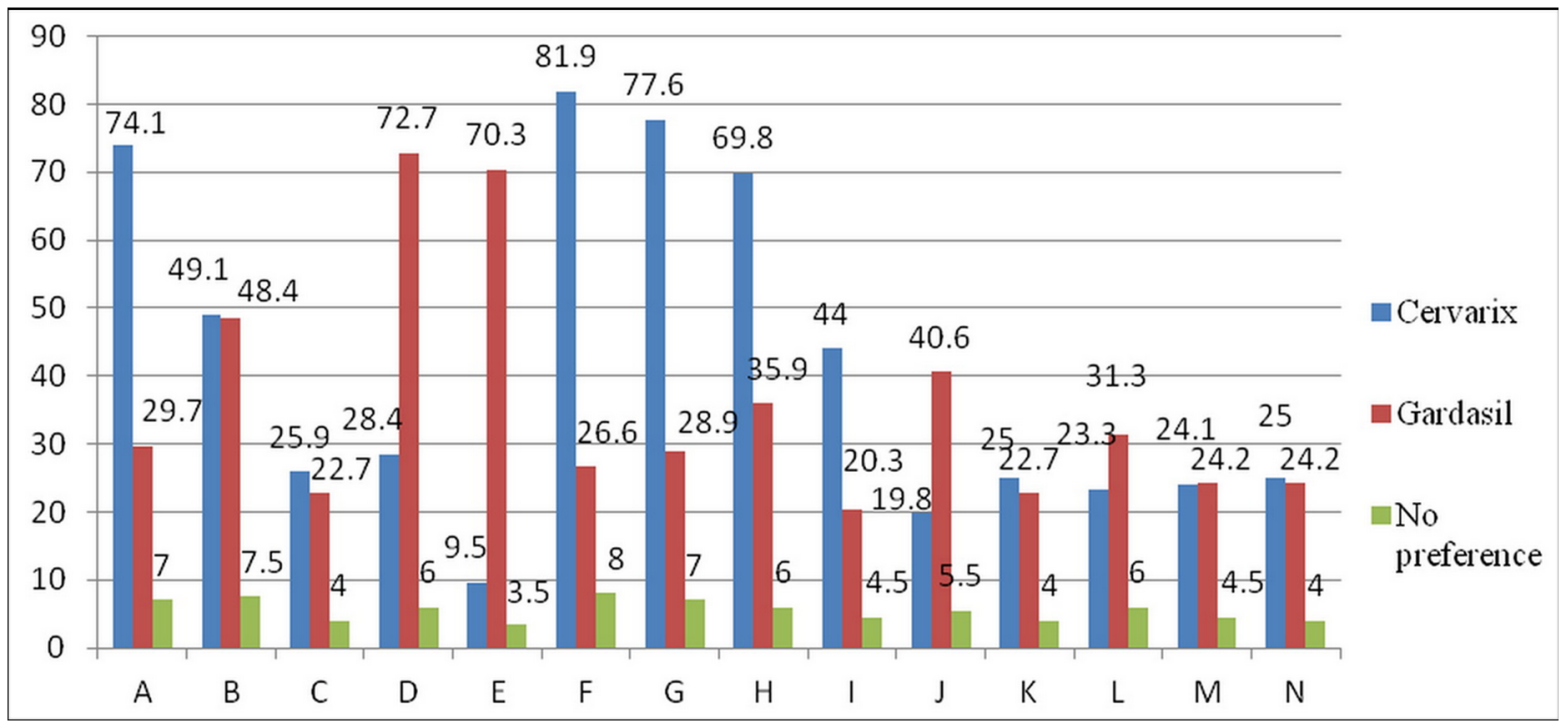
The assoication between preferred vaccine (Cervarix® vs. Gardasil® vs. no preference) and the reasons to choose vaccines (A to N). (A: Stronger protection; B: Safety; C: Lower price; D: More HPV types; E: Protect against genital warts; F: Better antibody response; G: Long-lasting immunity; H: Cross-protection for other cancer-associated HPV types; I: Better adjuvant; J: Patients think it’s better; K: Practice management; L: Selected by the Government; M: Credibility of manufacturer; N: Comprehensive service of manufacturer). All comparisons among vaccine preference groups across reasons to choose vaccine categories (A to N) were statistically significant (p<0.05).

41.1% of participants did not express any preference to the two available vaccines (Table 3). Among those indicated a preference, Gardasil^®^ (30.9%) and Cervarix^®^ (28.0%) were approximately equally preferred. The major reason for choosing a vaccine regarded by the participants as “extremely important” or “very important” included its strength of protection (61.1%), immunity being long-lasting (56.8%), good antibody response (55.6%), more HPV types covered (53.5%), cross-protection for other cancer-associated HPV types (53.4%) and vaccine safety (51.2%). Factors which were less considered included vaccine price (27.9%), credibility of manufacturers (28%) and comprehensive service of manufacturers (29.2%) (Table 3). Among physicians who preferred Cervarix^®^, greater proportions regarded stronger protection, better antibody response and long-lasting immunity as extremely or very important when compared with those who preferred Gardasil^®^ (all p<0.05) (Fig. 2). Alternatively, physicians choosing Gardasil^®^ had significantly higher proportions regarding more HPV types to be covered, its protection against genital warts, better patient perception, and Government selection (though our Government did not express any preference) as important reasons for the choice (all p<0.05) (Fig. 2).

**Table 3:** Preference and reasons to choose human papillomavirus (HPV) vaccines (bivalent vs. quadrivalent) in practice for adolescent girls aged 10–17 years.

	n		%^a^		
Bivalent - Cervarix®	116		28.0		
Quadrivalent - Gardasil®	128		30.9		
No preference	170		41.1		
	**Extremely or very important**	**Important**	**Fairly important**	**Somewhat important**	**Not at all important**
Stronger protection	138 (61.1)	49 (21.7)	14 (6.2)	8 (3.5)	17 (7.5)
Safety	134 (51.2)	79 (30.2)	23 (8.8)	8 (3.1)	18 (6.9)
Lower price	67 (27.9)	73 (30.4)	39 (16.3)	18 (7.5)	43 (17.9)
More HPV types	138 (53.5)	53 (20.5)	19 (7.4)	17 (6.6)	31 (12.0)
Protect genital warts	108 (44.6)	51 (21.1)	23 (9.5)	21 (8.7)	39 (16.1)
Better antibody response	145 (55.6)	63 (24.1)	23 (8.8)	12 (4.6)	18 (6.9)
Long-lasting immunity	141 (56.8)	63 (25.4)	19 (7.7)	8 (3.2)	17 (6.9)
Cross-protection for other cancer-associated HPV types	139 (53.4)	78 (30.0)	18 (6.9)	7 (2.7)	18 (6.9)
Better adjuvant	86 (34.4)	80 (32.0)	36 (14.4)	12 (4.8)	36 (14.4)
Patients think it’s better	86 (33.4)	73 (28.4)	36 (14.0)	20 (7.8)	42 (16.3)
Practice management	66 (26.8)	78 (31.7)	27 (11.0)	15 (6.1)	60 (24.4)
Selected by Government	79 (30.6)	88 (34.1)	29 (11.2)	19 (7.4)	43 (16.7)
Credibility of manufacturer	68 (28)	85 (35.0)	29 (11.9)	22 (9.1)	39 (16.0)
Comprehensive service of manufacturer	68 (29.2)	74 (31.8)	27 (11.6)	24 (10.3)	40 (17.2)

a Percentages in brackets were valid % which excluded missing variables.

### Perceived Barriers to Advise Adolescents Aged 10–17 Years for HPV Vaccination

The most frequent factors perceived as “extremely likely” or “somewhat likely” barriers to advise HPV vaccination among adolescents included parental refusal due to safety concerns (78.6%), parental reluctance to discuss sexuality and sexually transmitted diseases (70.4%), and physicians being regarded as hard selling an expensive vaccine (65.0%) (Table 4). Most perceived that parents were less attentive to vaccines once the child was older than 5 years (62.6%). The participating physicians also concerned that parents would refuse based on too many vaccines for their children (56.1%), or parental disbelief in vaccination (56.8%). Physicians also perceived the difficulty to get adolescents showing up for well visits (58.0%), too low parental awareness or being time-consuming (51.8%), as well as parental perception of labeling their children at risk for sexually transmitted diseases (44.9%), were extremely or somewhat important barriers (Table 4).

**Table 4:** Perceived barriers to advising adolescents aged 10–17 years for human papillomavirus (HPV) vaccination.

	Extremely Likely or Somewhat Likely	Extremely Unlikely, Somewhat unlikely, or Neutral
**Parental refusal**		
Child too many vaccine	198 (56.1)	155 (43.9)
Safety concern	282 (78.6)	77 (21.4)
Not believe in vaccine	201 (56.8)	153 (43.2)
**Less attentive to vaccine once child is >5 years**	229 (62.6)	137 (37.4)
**Parental reluctance to discuss sexuality/STD**	254 (70.4)	107 (29.6)
**Parental perception of singling out their child as** **at risk for STD**	164 (44.9)	201 (55.1)
**Parental concern of increasing risky behavior**	149 (41.0)	214 (59.0)
**Frequent changes of recommendation for** **immunization**	155 (42.9)	206 (57.1)
**Doctors’ reluctance to offer multiple vaccines**	107 (30.1)	249 (69.9)
**Doctors’ reluctance to discuss sexuality/STD**	118 (32.3)	247 (67.7)
**Difficult to get adolescents to show up for well** **visits/immunizations**	207 (58.0)	150 (42.0)
**Doctors’ reluctance since regular pap smear is still** **needed after vaccine**	96 (26.4)	268 (73.6)
**Doctors’ reluctance as vaccine’s protection is <100%**	102 (28.2)	260 (71.8)
**Too low parental awareness, time-consuming**	187 (51.8)	174 (48.2)
**Hard selling an expensive vaccine**	234 (65.0)	126 (35.0)
**Vaccination not related to the reasons of** **consultation**	201 (55.7)	160 (44.3)
**Not the doctors’ responsibility**	65 (18.5)	286 (81.5)
**Difficulty to initiate the conversation**	125 (34.4)	238 (65.6)
**Most adolescent patients not at risk for** **HPV infection**	86 (24.2)	269 (75.8)
**Most adolescent patients not at risk for cervical** **cancer**	85 (23.5)	277 (76.5)

STD: Sexually Transmitted Disease.

### Practice of HPV Vaccination According to Recommendations, Age Groups and Sex

A majority of the physicians reported that the recommendations to prescribe HPV vaccine to adolescents were “extremely likely” or “somewhat likely” influenced by the local Government (the Department of Health) (95.8%), the respective colleges of their specialties (93.8%), local University academics (90.7%) and overseas authorities or colleges (86.8%) (Table 5). Very few physicians recommended HPV vaccine among males irrespective of patients’ age and the nature of vaccine protection (Table 5). A substantial proportion recommended HPV vaccines among females aged 18–26 years for prevention of cervical cancer alone (95.7%), and genital warts and cervical cancer combined (96.9%). The proportions were similarly high among females aged 10–17 years and 27–36 years, whilst the proportion recommending HPV vaccine was fewer among women aged 37–45 years (Table 5).

**Table 5:** Practice of human papillomavirus (HPV) vaccination according to recommendations, age groups and sex.

	Extremely Likely or Somewhat Likely to follow recommendation	Extremely Unlikely, Somewhat unlikely, or neither likely or unlikely to follow recommendation
**Your colleagues**	346 (83.6)	68 (16.4)
**The Hong Kong College of your specialty**	393 (93.8)	26 (6.2)
**The Department of Health**	415 (95.8)	18 (4.2)
**The oversea authorities or colleges**	363 (86.8)	55 (13.2)
**Local university academics**	380 (90.7)	39 (9.3)
**A pharmaceutical representative**	285 (68.0)	134 (32.0)
**Cervical cancer only**		
10–17 year old girl	403 (94.8)	22 (5.2)
10–17 year old boy	141 (34.0)	274 (66.0)
18–26 year old girl	402 (95.7)	18 (4.3)
18–26 year old boy	132 (32.4)	275 (67.6)
27–36 year old female	387 (92.8)	30 (7.2)
27–36 year old male	113 (27.6)	296 (72.4)
37–45 year old female	352 (84.4)	65 (15.6)
37–45 year old male	105 (25.4)	308 (74.6)
**Genital warts and cervical cancer**		
10–17 year old girl	408 (96.9)	13 (3.1)
10–17 year old boy	281 (68.2)	131 (31.8)
18–26 year old girl	405 (96.9)	13 (3.1)
18–26 year old boy	279 (68.4)	129 (31.6)
27–36 year old female	394 (94.9)	21 (5.1)
27–36 year old male	242 (59.5)	165 (40.5)
37–45 year old female	361 (87.0)	54 (13.0)
37–45 year old male	219 (53.2)	193 (46.8)

## Discussion

### Major Findings

This study found that the knowledge on HPV prevalence was low yet the knowledge on the role of different HPV types in cervical cancer and anogenital warts was high among PCPs in Hong Kong. They regarded HPV vaccine relatively important for adolescent health, and should preferably be administered at the age range of 12–14 years. Cervarix^®^ and Gardasil^®^ were almost equally preferred, with the major factors for vaccine choice being their strength of protection, duration of immunity, cross-protection for other cancer-related HPV types, types of HPV covered and safety. Parental refusal based on vaccine safety and reluctance to discuss sexually transmitted diseases as perceived by physicians represented the most frequently encountered barriers. Most physicians recommended HPV vaccination among females aged 18–26 years, and followed guidelines from the Government and their respective colleges.

### Compared with Other Studies - knowledge

A survey among 214 Vaccine For Children (VFC) providers in Georgia reported high levels of knowledge about the epidemiology of HPV infection, the causative role of HPV for cervical cancer, and the status of FDA approval for quadrivalent HPV vaccines for use in adolescents aged 9–26 years (range 76.7% to 97.2%) [23]. In addition, one multi-centre survey involving 1,282 Canadian physicians also showed that their knowledge about causation of HPV to cervical cancer and anogenital cancer was high (56.2% to 96.1%) [29]. On the contrary, a large-scale cross-sectional study among a nationally representative sample of primary care pediatricians in US showed that almost one-third were unaware that HPV has a causative role in cervical cancer [21]. A recent survey in West Yorkshire among 222 general practitioners, pediatricians and gynecologists showed that 38% felt inadequately informed about the HPV vaccine, and up to 55% had lack of knowledge about the aetiology of cervical cancer [28]. Hence, current literature pointed towards heterogeneity of knowledge levels among physicians depending on the location of survey and their specialties. Our study pointed towards lower levels of knowledge on the epidemiology of HPV infection but higher levels of knowledge on etiology when compared with these surveys.

### Compared with Other Studies - Attitude

Turning to physicians’ attitude towards HPV vaccine, a high degree of overall acceptance for HPV vaccine use directed against cervical cancer and genital warts was consistently reported by several large-scale studies, including primary care providers like family physicians, gynecologists and pediatricians [21], [22], [24]–[29]. A recent systematic review [33] including 25 studies showed strong support for HPV vaccination in their practice although gynecologists did not perceive themselves as traditional vaccinators [22]. These were compatible with our findings that PCPs perceived HPV vaccine was an important strategy to promote adolescent health. Of interest is the discrepancy between the ideal age range (12–14 years) for HPV vaccination perceived by PCPs and the age group (18–26 years) most commonly recommended for vaccination. This may reflect some underlying barriers preventing PCPs to recommend vaccination to young teenagers despite their wish to do so.

### Compared with Other Studies – Practice and Barriers

For the ideal age of adolescent girls to receive HPV vaccine from the PCPs’ perspective, the present study reported 12–14 years as the range most commonly selected by our study participants. One study reported that 89% of pediatricians in the US recommended HPV vaccines to girls aged 16–18 years, whilst only 46% recommended vaccines for adolescent girls aged 10–12 years [21]. Similarly, another study in US showed that fewer family physicians and pediatricians recommended HPV vaccines for children aged 11–12 years than for older female subjects [25]. The current US Food and Drug Administration approved quadrivalent HPV vaccine to be used for females aged 9 to 26 years in 2006 [34], and the Advisory Committee on Immunization Practices (ACIP) voted to administer HPV vaccines among adolescents aged 11–12 years [35]. Our study found that a large proportion of PCPs perceived 10–11 years (31.6%) and 12–14 years (45.6%) as the ideal age ranges. This is compatible with recommendations from international authorities. Of note is the relatively high proportion of PCPs who were influenced by recommendations by pharmaceutical representatives. One possible reason is the high level of drug marketing activities in the primary care sector of Hong Kong, and that most PCPs develop a good relationship with drug salespersons who become more influential on prescription practices.

From a recent systematic review [33], cost of vaccine and/or problems with its reimbursement, providers’ and parental concern about vaccine safety and efficacy, perception of clients being too young for HPV vaccination, the potential disadvantages of HPV vaccine as promoting sexual activities, organizational influence, communication related to sexuality, and lack of education were identified as major barriers of HPV vaccine prescriptions. Our study findings on barriers were in general similar with current literature.

### Implications to Clinical Practice and Future Research

This study has several important implications. Firstly, more educational initiatives should be organized for PCPs working in the private sector so as to enhance their perceived importance of prescribing HPV vaccine to their clients and patients. The focus should be on the epidemiology of HPV infection and genital wart, as well as proportion of cervical cancer attributed to HPV types covered by current vaccines. This should be well received as PCPs strongly agreed that HPV vaccine has a higher priority in improving adolescent health as compared with other vaccines. Several aspects in these educational initiatives should be emphasized, including the ability of HPV vaccines to achieve strong protection with long-lasting immunity, some degrees of cross-protection against the important oncogenic types of HPV, and their good safety profiles. Secondly, since parental refusal was mainly on the vaccine’s safety, parents should be particularly reassured about its low rates of adverse effects in lieu of its many health benefits. The parents’ concerns on discussing sexuality and sexually transmitted diseases signify the crucial role played by PCPs in adopting a holistic approach in their consultations, lending higher levels of attention to discuss sensitive issues. Consultations with prospective clients and patients eligible to receive HPV vaccine should be cautiously conducted with good communication skills taking possible embarrassment to girls and their parents into account. Also, since the Governmental recommendations and the PCPs’ respective academic colleges were perceived as the most influential in affecting their practice of prescribing HPV vaccine, they should be the primary agents to deliver educational messages. That a high proportion of PCPs suggesting full subsidy of the vaccine by the Government also implied that the vaccine might raise an affordability issue. At the early stage to enhance vaccine uptake rate among adolescent girls, the Government should consider at least partial subsidy to eligible clients to overcome vaccine cost which is one of the important barriers.

In light of the present findings, future research should evaluate interventions which could effectively enhance PCPs’ knowledge of HPV and its vaccination in a sustainable manner; remove the barriers of offering immunization, and optimize proper practice of HPV vaccines in suitable subjects.

### Strengths and Limitations

To our knowledge this study is the first-ever evaluation among Chinese PCPs on their prescriptions of HPV vaccination. The validity of survey instrument, the high response rate and the sampling methodology enhanced the generalizability of the findings. However, several limitations should be addressed here. Firstly, this is a cross-sectional study and one could not establish causal relationships between PCPs’ knowledge or attitude and their HPV prescriptions. In addition, we had invited PCPs working in the private sector only, and the attitudes and barriers of HPV vaccine prescription might differ to a certain extent with physicians working in the public sector and also doctors in the academic field; the PCPs were heterogeneous with respect to their specialties and we have adopted an aggregate analysis. Also, since the demographic and practice characteristics of all PCPs in Hong Kong are not known, the generalizability of the present findings could not be ascertained. Furthermore, this is a descriptive analysis and there could be potential confounders affecting clinical practice, like the choice between Gardasil and Cervarix. Lastly, this cross-sectional study was conducted in 2011–2012, five years after the licensure of the HPV vaccines in Hong Kong, and it is anticipated that the perceptual variables pertinent to the vaccine will change with time as more promotional materials and media communications emerge. A time series of cross-sectional surveys is needed to reflect the trends of changing knowledge, attitude and perceived barriers.

## Conclusions

There is still room to equip PCPs the knowledge on HPV epidemiology infection and skills in motivating behavioural change in discussing sex related issues not directly related common presentations by adolescents. Public health education on safety and efficacy of the HPV vaccination is still needed. Recommendation by Government including subsidy would be an important factor to enhance vaccination rates.

## What Is Already Known on This Topic

Human papillomavirus (HPV) infection could be effectively prevented by vaccination, and physician recommendation has been known to be the most influential factor for patients’ uptake of the vaccine. However, few studies explored the knowledge, attitude, barriers and practice of HPV vaccination among primary care physicians.

## What This Study Adds

The knowledge of 444 physicians on HPV infection was low. Vaccination against HPV was perceived as more important than other vaccines, and Government subsidy was regarded as an important public health strategy. The most significant barriers to prescribe HPV vaccines consisted of parental refusal due to safety concerns. Public health education on safety and efficacy of vaccination is needed, and support by Governmental funding would be an important factor to enhance vaccination rates.
